# miR-19-3p Targets PTEN to Regulate Cervical Cancer Cell Proliferation, Invasion, and Autophagy

**DOI:** 10.1155/2023/4784500

**Published:** 2023-03-03

**Authors:** Wei Wang, Lu Liu, Yongjian Tian

**Affiliations:** ^1^Medical Laboratory, Fuyang City People's Hospital, Fuyang 236004, Anhui, China; ^2^College of Veterinary Medicine, Xinjiang Agricultural University, Urumqi 830052, Xinjiang, China

## Abstract

**Background:**

Cervical cancer is the second most common cancer among women worldwide. Extensive studies have shown that microRNAs (miRNA/miR) can regulate the formation, progression, and metastasis of cancer. The purpose of this study was to investigate the effect of miR-19-3p on the proliferation, invasion, and autophagy of cervical cancer cells and to explore the underlying mechanism.

**Methods:**

SiHa and HeLa cells were transfected with miR-19-3p mimic and inhibitor. miR-19-3p and PTEN expression were detected using real-time quantitative PCR and western blot, respectively. The binding between miR-19-3p and PTEN was predicted using Targetscan7.2 and verified by a dual-luciferase reporter gene assay. The effects of miR-19-3p on cell invasion and proliferation were evaluated by Transwell assays and MTT, respectively. The effect of miR-19-3p on autophagy was observed using fluorescence microscopy.

**Results:**

The expression of miR-19-3p in cervical cancer tissues and SiHa and HeLa cells was significantly upregulated, whereas the expression of PTEN was significantly downregulated. PTEN was one of the direct targets of miR-19-3p. The miR-19-3p mimic significantly reduced the apoptosis rate and autophagy and promoted cell proliferation and invasion of the SiHa and HeLa cells.

**Conclusion:**

In summary, miR-19b-3p can target PTEN to regulate the proliferation, invasion, and autophagy of cervical cancer cells. Our findings indicate the potential of miR-19-3p as a target for cervical cancer treatment in the future.

## 1. Introduction

Cervical cancer is the second leading cause of cancer-related deaths among women worldwide [1, 2]. Persistent infection with high-risk human papillomavirus (HPV) is considered the main risk factor for cervical cancer [3]. About 50% of cervical cancers are related to persistent HPV16 infection, and about 20% are related to persistent HPV18 infection [4–6]. However, only ∼15% of women with persistent high-risk HPV infection will develop cervical cancer, which indicates that other factors are involved in the regulation of the occurrence and development of cervical cancer [7]. The HPV vaccine has no protective effect on patients who are already infected with HPV or have cervical lesions. Therefore, understanding the pathogenesis of cervical cancer is important for developing therapeutic targets for this type of cancer.

MicroRNAs (miRNA/miR) are small noncoding single-stranded RNAs (19–25 nucleotides in length) [8]. miRNA silences its target genes via base-pairing with complementary sequences of 3′-untranslated region (3′-UTR) in the mRNAs of the target gene, thereby inhibiting translation or inducing mRNA degradation [9, 10]. In this way, miRNA is involved in many biological processes, including cell proliferation, apoptosis, cell differentiation, and development [11–15]. Accumulating evidence supports the importance of miRNA in cervical cancer. For example, Wang et al. reported that miRNA signatures can be useful for the screening, diagnosis, and prognosis of cervical cancer [10, 16, 17]. Hu et al. and Park et al. reported that miR-200a, miR-9, and miR-944 can be used to predict the survival rate of cervical cancer [18, 19]. The aberrant expression of miR-466 and miR-34a is closely associated with the occurrence and development of cervical cancer [20, 21]. miR-21 can promote the proliferation of HeLa cells by targeting programmed cell death [22]. It has also been shown that miR-361 targets HSP90 to inhibit the invasion and epithelial-mesenchymal transition (EMT) of SiHa and HeLa cells [23]. There is increasing evidence about miRNA as a new biomarker and therapeutic target for cervical cancer. Upregulation and downregulation of miR-19b have been reported in various cancers, including rectal cancer [24], breast cancer [25], lung cancer [26], and pancreatic cancer [27], and miR-19b has been identified as a key regulatory molecule in the mechanism of cancer development. However, the role of miR-19b-3p in cervical cancer remains unknown.

In this study, SiHa and HeLa cells were transfected with miR-19-3p mimics and inhibitors to detect cell proliferation, invasion, and autophagy. The potential target of miR-19-3p was determined using the bioinformatics analysis tool TargetScan7.2 and verified using a dual-luciferase reporter assay. This study will clarify the molecular mechanism of miR-19b-3p in regulating the occurrence and development of cervical cancer. We present the following article in accordance with the MDAR reporting checklist.

## 2. Materials and Methods

### 2.1. Tissue Samples

In total, 20 pairs of cervical cancer tissues (tumour) and adjacent tissue (normal) samples were obtained from HPV16/HPV18-positive cervical cancer patients (age: 53.20 ± 10.21 years) who were admitted to Fuyang City People's Hospital from July 2019 to June 2020. For control, normal cervical exfoliated cells were collected from 10 healthy subjects who were HPV negative and without cervical lesions. The study was approved by the Institutional Ethics Board of Fuyang City People's Hospital (no. (2020)0026), and informed consent was obtained from all individual participants.

### 2.2. Cell Culture and Transfection

Human cervical cancer cell lines SiHa and HeLa were cultured with DMEM medium (11885092, Gibco, USA) containing 10% fetal bovine serum (FBS) (CCS30013.01HI, MRC, Australia), 1% penicillin/streptomycin (15140122, Gibco, USA), and 2 mM glutamine. The cells were then cultured in a 5% CO_2_ incubator at 37°C and were passaged when confluence reached 90%.

Next, the cells were transfected with miR-19-3p mimic (mimic), miR-19-3p mimic negative control (mimic-NC), miR-19-3p inhibitor (inhibitor), and miR-inhibitor negative control (inhibitor-NC), which were all obtained from Sangon Biotech (Shanghai) Co., Ltd., using Lipofectamine 2000 (11668019, Invitrogen, USA).

### 2.3. Cell Proliferation Assay

The cells were seeded in a 96-well plate (4 × 10^4^ cell/well) and cultured at 37°C with 5% CO_2_. At 24, 48, and 72 h of culture, 10 *μ*l of MTT solution (5 mg/ml, Sigma, USA) was added to each well and incubated at 37°C for 4 h. Dimethyl sulfoxide (DMSO) (150 *μ*l/well) was added to dissolve the formazan crystals at 37°C for 30 min. Finally, a Multiskan™ FC Microplate Photometer (Multiskan FC, Thermo Fisher, USA) was used to measure the absorbance at 450 nm.

### 2.4. Transwell Invasion Assay

The serum-free cell suspension (5 × 10^5 ^cells/ml; 200 *μ*l) was added to the Transwell upper chamber, and 500 *μ*l of DMEM medium containing 10% FBS was added to the lower chamber (Corning, USA). The Transwell chambers were cultured at 37°C and 5% CO_2_ for 24 h. Then, the cells were fixed with 4% paraformaldehyde for 15 min and stained with 0.1% crystal violet. Next, the cells were observed under an inverted fluorescence microscope (Axio Observer, Zeiss, Germany), and the migrated cells were counted.

### 2.5. Real-Time Quantitative PCR (RT-qPCR)

Total RNAs were extracted using TRIzol (10296010, Invitrogen, USA). The reverse transcription of RNA into cDNA was performed using a PrimeScript™ RT reagent kit (RR037A, Takara, Japan). We used a Mir-X miRNA first-strand synthesis kit (638315, Takara, Japan) to detect the expression of miR-19-3p, and U6 was the internal reference gene. Using the TB Green® Premix Ex Taq™ II kit (RR820 B, Takara, Japan), we detected the expression of *MUC4* mRNA, with *GAPDH* being the internal reference gene. The primers were purchased from Sangon Biotech (Shanghai) Co., Ltd (Table 1). The PCR procedure was 95°C for 5 min followed by 40 cycles at 95°C for 15 s and 60°C for 30 s. The reaction was performed using a fluorescence quantitative PCR instrument (AFD4800, AGS, Hangzhou, China). The relative expression levels were calculated using the 2^−ΔΔCt^ method [28].

### 2.6. Western Blot Analysis

RIPA Lysis Buffer (BL504A, Biosharp) was used to extract the total protein from the cervical cancer tissues and cells, and the protein concentration was determined by BCA (BL521A, Biosharp). The protein sample (40 *μ*g) was subjected to 10% SDS-PAGE. The protein was then transferred to the PVDF membranes. After blocking with 5% BSA (4240GR025, Biofroxx) for 2 h at room temperature, the membranes were incubated with anti-PTEN (1 : 1000, ab32199, Abcam) and anti-GAPDH (1 : 1000. ab9485, Abcam) antibodies at 4°C overnight. After washing 3 times with TBST for 5 min each time, incubation with HRP-labelled secondary antibody (1 : 2000 dilution, ZB-2301, ASGB-BIO, China) was performed for 1 h at 37°C. Enhanced chemiluminescence (PE0010, Solarbio) was used for colour development. The protein bands were then analysed in Image J (V1.52s, Bharti Airtel Ltd.).

### 2.7. Acridine Orange and Propidium Iodide Staining

After staining with acridine orange (AO), red and yellow acid bodies were observed under a fluorescence microscope. SiHa and HeLa cells were collected and stained with AO and propidium iodide (PI) (5 *μ*g/ml). An inverted fluorescence microscope was used to observe and analyse autophagy through the FITC/TRITC channel under 20x and 40x objective lenses [29, 30].

### 2.8. Dual-Luciferase Reporter Gene Assay

The target genes of miR-19-3p were predicted using TargetScan7.2 (https://www.targetscan.org/vert_71/). The 3′-UTR of *PTEN* mRNA containing the putative miR-19-3p binding site was amplified by PCR, and the resulting products were cloned into the pmirGLO dual-luciferase vector (E1330, Promega, USA). Site-directed mutagenesis on the predicted target site of miR-19-3p in wild-type (WT) *PTEN* 3′-UTR was conducted using a QuickChange II site-directed mutagenesis kit (200521, Agilent Technologies, Inc., Santa Clara, CA, USA). A *PTEN* 3′-UTR mutation (MUT-*PTEN* 3′-UTR) reporter gene plasmid was constructed. A Lipofectamine 2000 kit (Invitrogen, 11668019, USA) was used to cotransfect the reporter gene plasmid and miR-19-3p mimics or miR-19-3p inhibitor into cells. At 48 h after transfection, luciferase activity was determined using a dual-luciferase reporter assay system (E2920, Promega, USA) according to the manufacturer's instructions.

### 2.9. Statistical Analysis

SPSS 18.0 (IBM Corp., Armonk, NY, USA) was used for the statistical analysis. The data were expressed as the mean ± standard deviation (mean ± SD). A two-tailed *t*-test was used to compare the differences between the two groups. A one-way ANOVA was used to compare differences among multiple groups. The correlation between two variables was analysed using Pearson correlation. *P* < 0.05 was considered to be significant [31].

## 3. Results

### 3.1. Upregulation of miR-19-3p and downregulation of *PTEN* in Cervical Cancer

**Figure 1:**
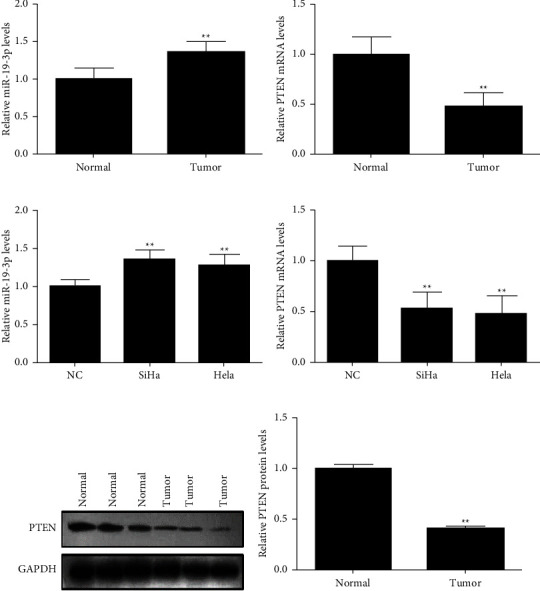
Expression of miR-19-3p and PTEN in cervical cancer tissues and cells. (a) Relative expression of miR-19-3p in tumour tissues (tumour) and adjacent tissues (normal). (b) Relative expression of *PTEN* in the tumour and normal tissues. (c) Relative expression of miR-19-3p in SiHa, HeLa, and normal cervical exfoliated cells (NC). (d) Relative expression of *PTEN* mRNA in SiHa, HeLa, and NC. (e, f) Expression of PTEN protein in the tumour and normal tissues. ^*∗*^*P* < 0.05, ^*∗∗*^*P* < 0.01.

The relative expressions of miR-19-3p and *PTEN* mRNA were detected using RT-qPCR. Our results showed that the relative expression of miR-19-3p in the tumour samples was significantly higher than in the normal samples (*P* < 0.01) (Figure 1(a)), while the relative expression of *PTEN* mRNA in the tumour samples was significantly lower than in the normal samples (*P* < 0.01) (Figure 1(b)). For the expression of miR-19-3p and *PTEN* in SiHA, HeLa, and NC cells, the results showed that the relative expression of miR-19-3p in SiHa and HeLa cells was significantly higher than in the NC cells (*P* < 0.01) (Figure 1(c)). However, the relative expression of *PTEN* mRNA in the SiHa and HeLa cells was significantly lower than that in the NC cells (*P* < 0.01) (Figure 1(d)). The expression of PTEN protein in tumour and normal samples was detected by western blot. The results showed that the expression of PTEN protein in tumour samples was significantly lower than in normal samples (*P* < 0.01) (Figures 1(e) and 1(f)). These results suggest that miR-19-3p is upregulated and PTEN is downregulated in cervical cancer.

### 3.2. The Proliferation and Invasion of Cervical Cancer Cells are Regulated by miR-19-3p

**Figure 2:**
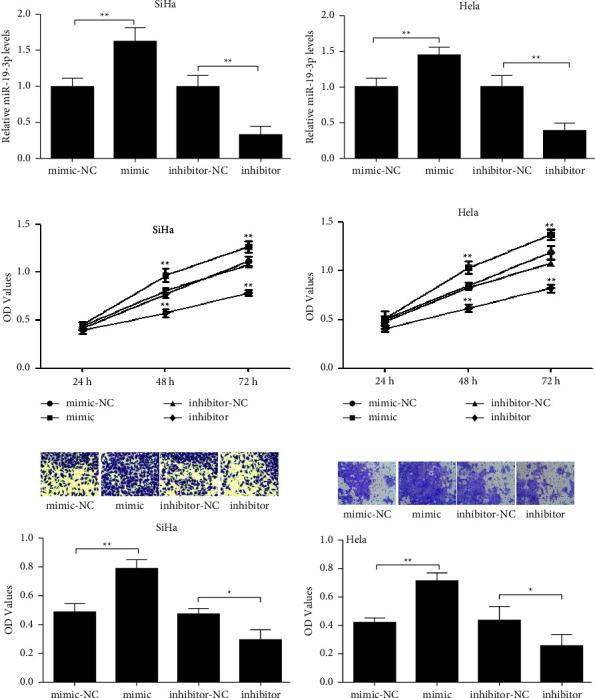
Proliferation and invasion of SiHa and HeLa cells after miR-19-3p mimic and inhibitor transfection. (a, b) Relative expression of miR-19-3p after miR-19-3p mimic and inhibitor transfection in SiHa and HeLa cells. (c, d) Cell proliferation of SiHa and HeLa cells after transfection. (e, f) Cell invasion of SiHa and HeLa after transfection. ^*∗*^*P* < 0.05, ^*∗∗*^*P* < 0.01.

The mimics and inhibitors were transfected into SiHa and HeLa cells. The relative expression of miR-19-3p was detected using RT-qPCR. Cell proliferation was detected with an MTT assay, and cell invasion was detected with a Transwell assay. The results showed that the miR-19-3p expression, cell proliferation, and invasion after transfection showed the same trend in the SiHa and HeLa cells. The relative expression of miR-19-3p in the mimic group was significantly higher than that of the mimic-NC group (*P* < 0.01), while the relative expression of miR-19-3p in the inhibitor group was significantly lower than in the inhibitor-NC group (*P* < 0.01) (Figures 2(a) and 2(b)). The proliferation of SiHa and Hela was significantly upregulated at 48 h after mimic transfection (*P* < 0.01) but was significantly downregulated at 48 h and 72 h after transfection with inhibitor (*P* < 0.01) (Figures 2(c) and 2(d)). The invasion of the SiHa and HeLa cells was significantly inhibited at 48 h after transfection with the inhibitor (*P* < 0.05) (Figures 2(e) and 2(f)). These results suggest that miR-19-3p regulates the proliferation and invasion of cervical cancer cells.

### 3.3. Cervical Cancer Cell Autophagy is Regulated by miR-19-3p

**Figure 3:**
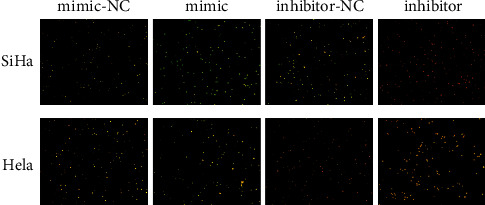
The effect of miR-19-3p on the autophagy of SiHa and HeLa. The intensity of red fluorescence reflects the degree of autophagy.

The degree of autophagy was observed using fluorescence microscopy after miR-19-3p mimic transfection. The results showed that SiHa and HeLa in the mimic, mimic-NC, and inhibitor-NC groups mainly exhibited green fluorescence, and there was little red fluorescence in the cytoplasm and nucleus, suggesting that there was virtually no autophagy. The cells in the inhibitor group showed obvious red fluorescence, indicating that a large number of acidic autophagic lysosomal vacuoles had formed and that early apoptosis was induced (Figure 3).

### 3.4. miR-19b-3p Targets *PTEN*

**Figure 4:**
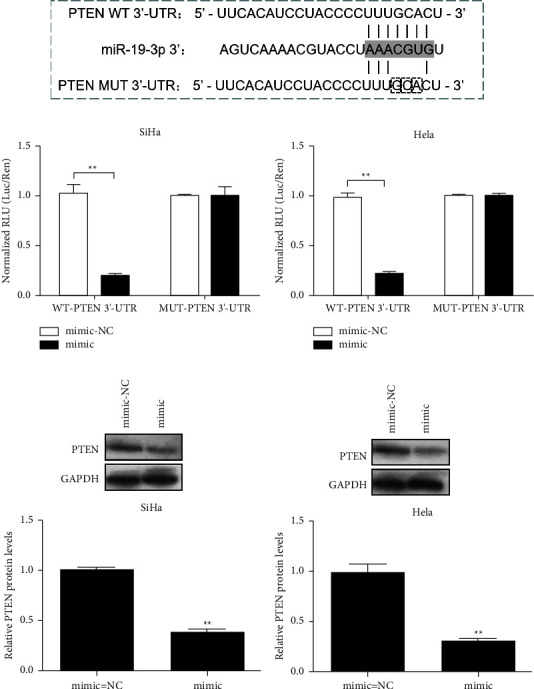
*PTEN* is the direct target of miR-19-3p. (a) The target site of miR-19-3p in *PTEN* 3′-UTR. The box indicates the mutation site; (b, c) luciferase reporter analysis of cells transfected with the WT or MUT-*PTEN* 3′-UTR reporter vector; (d, e) western blot was used to detect the expression of PTEN protein in SiHa and HeLa after miR-19-3p mimic transfection. ^*∗*^*P* < 0.05, ^*∗∗*^*P* < 0.01.

TargetScan7.2 was used to predict the target genes of miR-19b-3p. *PTEN* was identified as a potential target of miR-19-3p. The putative targeting sites of miR-19-3p in *PTEN* 3′-UTR are shown in Figure 4(a). The dual-luciferase reporter gene assay showed that the miR-19-3p mimic attenuated the luciferase activity of WT-*PTEN* 3′-UTR in SiHa and HeLa but had no significant effect on MUT-*PTEN* 3′-UTR (*P* < 0.01) (Figures 4(b) and 4(c)). Western blot was used to detect the regulation of PTEN by miR-19-3p mimics in SiHa and HeLa. The results showed that the miR-19-3p mimic significantly downregulated PTEN protein expression in SiHa and HeLa (*P* < 0.01) (Figures 4(d) and 4(e)). These results suggest that *PTEN* is the direct target of miR-19-3p.

## 4. Discussion

There has been increasing evidence that dysregulated miRNAs are involved in the regulation of a variety of physiological processes in tumours, including cell proliferation, drug resistance, apoptosis, metastasis, and angiogenesis [10, 32, 33]. Research on miRNAs focuses on cancer-specific up- or downregulated miRNAs and their target genes in order to clarify the pathogenesis of cancer [34, 35]. Numerous reports have shown that miR-19 positively regulates tumorigenesis, EMT, cancer cell proliferation and invasion, and metastasis by targeting different targets in rectal cancer [24], breast cancer [25], pancreatic cancer [26], osteosarcoma [36], and lung cancer [27, 37], indicating that miR-19 plays a carcinogenic role in cancer development. Our research confirmed that miR-19-3p is upregulated in cervical cancer and that miR-19-3p upregulation *in vitro* promotes the proliferation and invasion of SiHa and HeLa cells. These results indicate that miR-19-3p exerts a carcinogenic effect in cervical cancer. The target of miR-19-3p was further predicted and analysed to determine the potential mechanism of miR-19-3p in the occurrence and development of cervical cancer.

The *PI3K/AKT* pathway plays a vital role in cancer development. As a tumour suppressor gene, *PTEN* is a key negative regulator of the *PI3K/AKT* pathway, which is involved in regulating cell biological processes such as cell proliferation, invasion, and cycle arrest [38, 39]. Downregulation of PTEN expression in a variety of cancers has been reported [40–42]. PTEN is the downstream target of many miRNAs. For example, Chai et al. predicted that the potential target of miR-498 is *PTEN* and identified that miR-498 is overexpressed in triple-negative breast cancer cells and downregulated PTEN by directly binding to the 3′-UTR of *PTEN* mRNA [43]. Wang et al. reported that miR-301 regulates cell proliferation and invasion by regulating the expression of PTEN in oesophageal carcinoma [44]. Cao et al. reported that inhibition of miR-144-3p expression can upregulate PTEN expression and affect cell growth and metastasis [45]. In this study, it was predicted that using TargetScan7.2 that *PTEN* is a target gene of miR-19-3p, and a dual-luciferase reporter gene assay further confirmed that miR-19-3p could bind specifically to the 3′-UTR of *PTEN*. We further found that the miR-19-3p mimics upregulated miR-19-3p in SiHa and HeLa cells and inhibited the expression of PTEN protein. However, the miR-19-3p inhibitor reversed the inhibitory effect on *PTEN*. These results indicate that miR-19-3p can negatively regulate the expression of the target gene *PTEN*.

In summary, miR-19-3p can regulate the biological functions of cervical cancer cells by targeting *PTEN*. miR-19-3p is expected to become a potential therapeutic target for cervical cancer.

## 5. Conclusions

miR-19-3p downregulates the expression of PTEN by directly targeting the 3′-UTR of *PTEN* mRNA, thereby regulating the biological behaviours of cervical cancer cells, such as cell proliferation, invasion, and autophagy. This research provides new insights into the diagnosis and treatment strategies for cervical cancer.

## Figures and Tables

**Table 1:** Primer sequences for real-time quantitative PCR.

Primer	5′–3′
Human-*PTEN*-F	TGGATTCGACTTAGACTTGACCT
Human-*PTEN*-R	GGTGGGTTATGGTCTTCAAAAGG
Human-*GAPDH*-F	CTGGGCTACACTGAGCACC
Human-*GAPDH*-R	AAGTGGTCGTTGAGGGCAATG
Human-miR-19-3p-F	ACTGAGTCGTATCCAGTGCAA
Human-miR-19-3p-R	GTATCCAGTGCGTGTCGTGG
Human-*U6*-F	CTCGCTTCGGGCAGCACA
Human-*U6*-R	AACGCTTTCACGAATTTGCGT

## Data Availability

The data used to support the study are available from the corresponding author upon request.
